# PENYEK: Automated brown planthopper detection from imperfect sticky pad images using deep convolutional neural network

**DOI:** 10.1371/journal.pone.0208501

**Published:** 2018-12-20

**Authors:** Azree Nazri, Norida Mazlan, Farrah Muharam

**Affiliations:** 1 Faculty of Computer Science & Information Technology, UPM, Serdang, Malaysia; 2 Institute of BioScience, UPM, Serdang, Malaysia; 3 Faculty of Agriculture, UPM, Serdang, Malaysia; US Department of Agriculture, UNITED STATES

## Abstract

Rice is a staple food in Asia and it contributes significantly to the Gross Domestic Product (GDP) of Malaysia and other developing countries. Brown Planthopper (BPH) causes high levels of economic loss in Malaysia. Identification of BPH presence and monitoring of its abundance has been conducted manually by experts and is time-consuming, fatiguing and tedious. Automated detection of BPH has been proposed by many studies to overcome human fallibility. However, all studies regarding automated recognition of BPH are investigated based on intact specimen although most of the specimens are imperfect, with missing parts have distorted shapes. The automated recognition of an imperfect insect image is more difficult than recognition of the intact specimen. This study proposes an automated, deep-learning-based detection pipeline, PENYEK, to identify BPH pest in images taken from a readily available sticky pad, constructed by clipping plastic sheets onto steel plates and spraying with glue. This study explores the effectiveness of a convolutional neural network (CNN) architecture, VGG16, in classifying insects as BPH or benign based on grayscale images constructed from Euclidean Distance Maps (EDM). The pipeline identified imperfect images of BPH with an accuracy of 95% using deep-learning’s hyperparameters: softmax, a mini-batch of 30 and an initial learning rate of 0.0001.

## Introduction

Rice, a staple food in Malaysia and the most important crop in South East Asia, is being damaged by a rice planthopper complex which has now become a challenge to farmers at the national level. The rice planthopper complex has three species: the brown planthopper (BPH), *Nilaparvata lugens (Stal*), the whiteback planthopper, *Sogatella furcifera (Harvath*), and the leafhopper, *Nephotettix virescens*. The most destructive hopper among them is the BPH, which is considered as the most serious pest of rice in both temperate and tropical region of east and south Asia[1]. Pesticides are used to reduce the rice pest outbreaks caused by rice pests. However, the uncontrolled use of pesticide affects soil in long term. A pest management system needs to be devised in order to eliminate pests and reduce environmental contamination. Such a program requires that pest insects be counted and precisely targeted, which is a time-consuming task.

Monitoring is an important aspect of pheromone-based pest control [2], [3]. Traditional pest identification and counting has been done by the experts through visual recognition. Traditional methods include (1) tapping on stem, (2) counting on stem and (3) cage usage. Tapping on stem involves identification and counting pests on enamel plate after stooping and flapping the stems. The enamel plate is coated with glue to avoid the escape of planthoppers. The second technique is by using a sweep net to sweep the rice pests. The human expert then visually counts the number of rice pests in the cage or net. Counting directly on stem involves visually identifying the planthoppers and then counting the rice pests on the rice stems. Other pest monitoring systems have been developed that trap insect pests and capture the trap surface images digitally for subsequent analysis by human experts. Unarguably, these manual methods are time consuming and tedious. The low accurate rate typically occurs when the number of rice pests on the enamel plate is over fifty. At this stage, the experts estimate the number of the rice pests based on their personal experience.

Advances in machine learning have revolutionized computer vision and object recognition in general and in pest identification in particular [4]. Machine learning helps traditional methods of pest identification and counting by automating the process. Previous works have considered insect classification from image processing in overcoming humanly fallible including image acquisition settings and features which typically use insect specimens as image sources [5]–[10]. However, insect species have their own drawback in which they are well-preserved and imaged in an ideal laboratory environment. In addition, specimen images are consistent and captured at high resolution. Many experts have captured specimens in the wild to classify the images, yet still image them under laboratory conditions [4], [11–15]. The drawback in using this data acquisition is that image quality is typically worse than the specimen in laboratory case, although it can be controlled by imaging all the insects under a standard orientation or lighting.

Several pest identifications and classification systems have been proposed that based on machine learning. Image analysis with scene interpretation was proposed by Kumar et al. [16] by developing automatic detection of harmful insects in the greenhouse. The features extracted from the image are generated from three feature extraction methods: Gabor Filter, Pyramidal Histogram of Gradient and Colour data. The empirical results showed that the proposed system was able to detect 98.5% whiteflies (total was 1,283 whiteflies) and 91.8% greenfly (total was 49 greenfly). Support Vector Machine (SVM) was used as the classifier of choice. Early pest detection system using images captured by pan tile camera to detect and classify pests was proposed [17]. The images were recorded and delivered to a central server where the processing and the analysis were done. In the central server, the images were extracted and classified from video frames. Again, SVM was used to classify the images frame by frame. The aim of this system is to estimate the density of pests inside the greenhouse.

Another method used a network of cameras for the continuous survey of a greenhouse in estimating the population of pests. In this system, one camera was observing the sticky trap while the second was observing the plant and none flying insect pests [16]. The streaming images were analysed with a priori knowledge about the visual appearance of the detected insects. The classification and interpretation of the extracted features from images was done using neural learning and knowledge-based techniques. Image analysis techniques were used by Cho et al [18], who developed an automated identification of whiteflies, aphids and thrips in the greenhouse by using image analysis technique. The goal of the proposed system is quite comprehensive in which they aimed to accurately estimate the density of pests for pest management strategy and minimize the use of pesticides. Data acquisition was done by installing wireless cameras that continuously observe the sticky trap. The captured images are then sent to the cloud server to process and identify the extracted insect pests.

The bag-of-words approach and gradient-based features were proposed for developing a framework that can classify insect pests from the paddy field [19]. The data acquisition was done by collecting insect pest images from Google Images together with images taken by their faculty in the paddy fields. The images were regionalized using Scale-Invariant Feature Transform (SIFT) and Speed-Up Robust Features (SURF) descriptors. Codebooks were developed to map the descriptors into a fixed-length vector in histogram space and classify the feature histograms based on the Histogram of Oriented Gradient (HOG) descriptors and using SVM as the classifier. The accuracy of the classification was 90%. One study developed an automated identification and counting system for different insect pests captured with light-traps and proposed a novel segmentation method for middle-sized touching insects from an image [20]. Normalized cuts (NCuts) together with the optical flow angle was used to separate the touching insects according to the number of insects in each connected region. The proposed segmentation method was compared to k-means and watershed methods and achieved a better segmentation result.

Algorithmically, insect classification needs features to be recognized by classifiers that include wing structures [5]–[9], colour histogram features [21], [22], morphometric measurements [9], [10], [23], [24], local image features [21], [22], [25]–[28], and global image features [29]. The features extracted from insect images are classified by various machine learning algorithms include SVM [10]–[12][10], [12], [25], Artificial Neural Network (ANN) [26], [29], K-Nearest Neighbours (KNN) [26], [29]and ensemble methods [25][11], [26]. The identification of insect pests based on deep learning is still largely unexplored.

In trap-based pest monitoring especially with sticky pads, there are many challenges such as low image quality, inconsistencies derived from illumination, movement of the trap, movement of the pests, camera out of focus, the appearance of other objects, decay or damage to the insect, the presence of benign insects and many more. Among these challenges, the effects of decay or damage on insect images are still not yet overcome. Decayed or damaged insects have different outlines and morphology compared to intact insects. Sticky pads usually trap while they are flying towards the pad surface. The impact of the collision between the pad and the insects distort the body and morphology of insects. It is difficult to design an automated system that can identify distorted insect pests. Thus, a method for such a system is in urgent need. Various datasets have been utilized to push this area forward [30], [31]; yet decayed and damageed pest datasets are largely missing. Concerned by this gap in research, this study proposes an identification and classification of imperfect pest’s pipeline with CNN as the image classifier.

## Materials and method

This section describes the collection, curation and pre-processing of sticky pad images. Samples of insects were collected from the granary area Sawah Sempadan (3^o^27’55.94”N, 101^o^11’33.33”E), Tanjung Karang, Selangor, Malaysia (authority: Norida, M. 1—Institute Tropical agriculture and food security, Universiti Putra Malaysia. 2. Department of Agriculture Technology, Faculty of Agriculture, Unversiti Putra Malaysa). The paddy was about 70 days after planting and infested with BPH. Sticky glue was sprayed on plastic sheet which stick on the steel board plate. The plate was put on the base of paddy stem, where the BPH are usually located. Then, the stems were tapped for few times to ensure BPH were adhered on the glued. These methods were repeated few times at different paddy plant to get enough samples.

**Fig 1:**
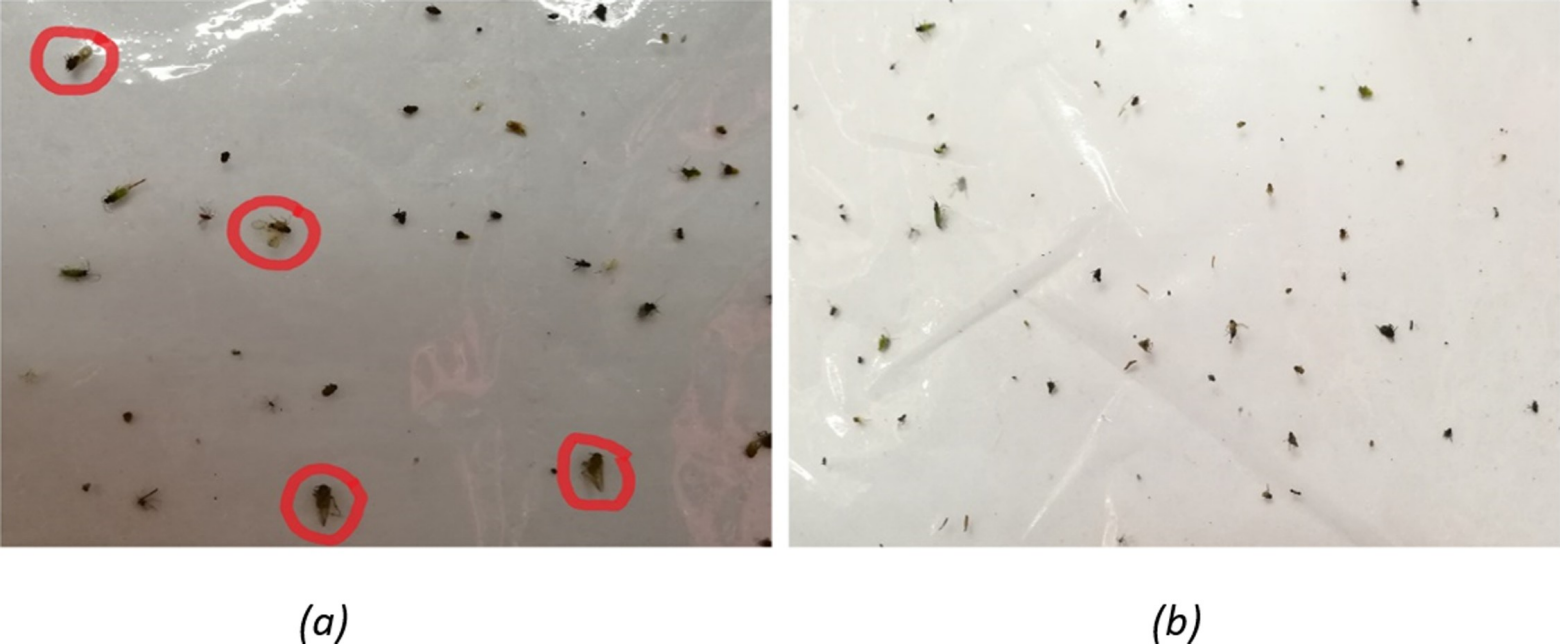
Examples of images captured from sticky trap. (a) trap with BPH and (b) trap with no BPH.

The plates, the sticky pad, consisting of trapped BPH were brought to a laboratory. The BPH, either short or long winged, were then manually identified and labelled with bounding circles by an entomologist. The digital images of the sticky pads were then captured by a Huawei P9 Plus smartphone camera under the laboratory room illumination and stored in JPEG format. The smartphone camera is dual 12 megapixels monochrome and colour camera, with focal length of 27mm and F2.2 aperture. All images do not have a temporal correlation with each other, which makes all the labelled BPH unique. Fig 1A shows a sticky pad image with all BPHs labelled with red bounding blue but cluttered with other types of insects while Fig 1B demonstrates an image containing benign insects (*Horvath*, *Nephotettix virescens* and other unidentified insects).

### Dataset construction

**Fig 2:**
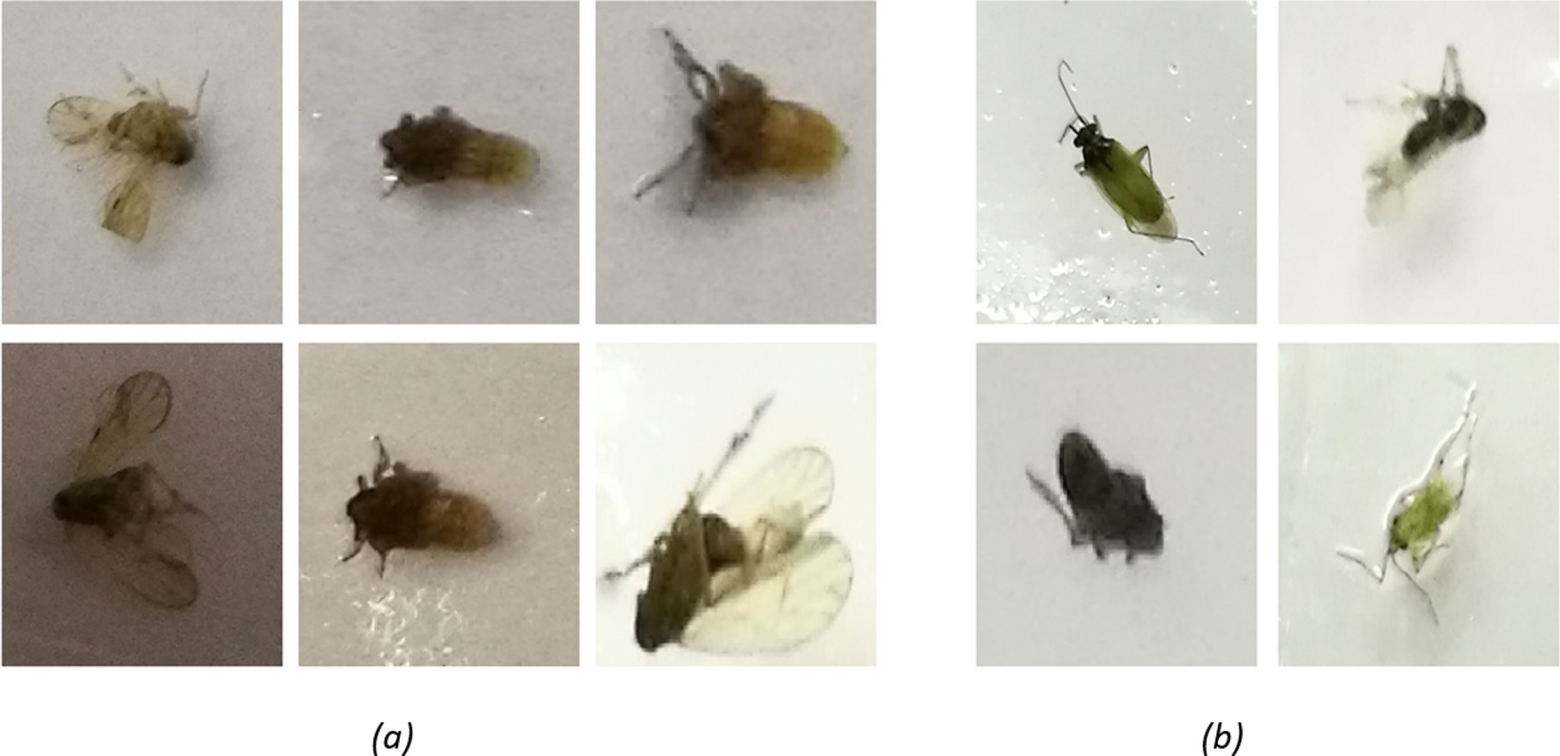
Examples of cropped images from sticky pad. (a) Positive patched containing a brown planthopper and (b) negative patches (benign).

Every single BPH and benign insect image was cropped from the sticky pad image to become an independent image patch. The positions and locations of these insects were random and unstructured. Fig 2A and 2B show BPH and benign images in their original position from the wild.

The dataset of both BPH and benign insects was split randomly into 3 sets: the training, validation and test sets. The statistics of each set is the same as the entire dataset as shown in Table 1.

**Table 1:** Statistics of constructed datasets.

Dataset	# images with BPH	# images with benign
Total	337	350
Training	236	245
Validation	34	35
Test	67	70

### Pre-processing

The sticky pad images were collected in real production environments which resulted in different imaging conditions at different times. As shown in Fig 2A and 2B, the most apparent challenge is illumination. To eliminate the potential negative effects of illumination variability on detection performance, the RGB image was transformed into a grayscale image [32].

### PENYEK

**Fig 3:**
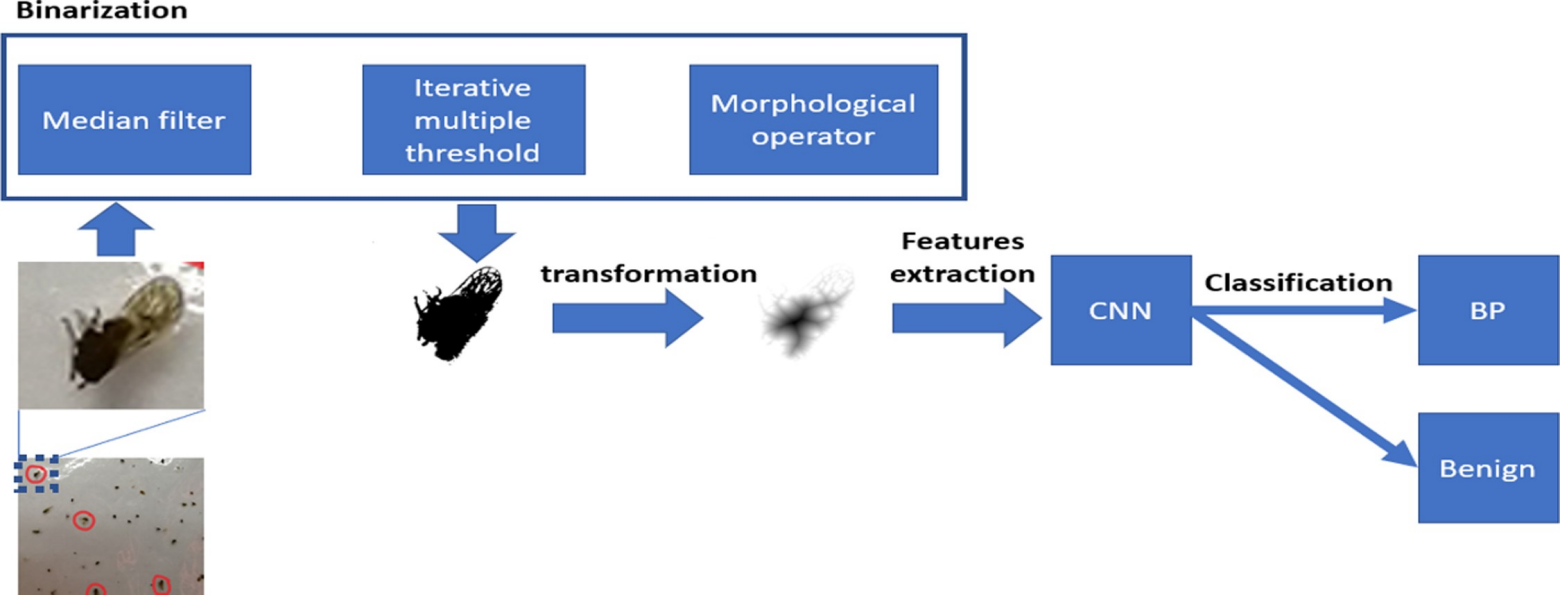
Illustration of the PENYEK classification pipeline.

A schematic diagram of PENYEK is shown in Fig 3. The proposed pipeline is called PENYEK because the word PENYEK means flatten object originated from the Malay language. The first step in the pipeline process was to crop the digital BPH images obtained from a sticky pad image. Median filtering was applied as a binarization step to reduce background noise and further to preserve the edges of the insect regions. The median filtering process replaces the centre value of the patch with the median value of all neighbouring pixel values. The second step in binarization process was the application of an iterative multiple thresholding algorithm to separate the image pixels into the foreground and background. The threshold estimation depends on the maximization of the between-class variances of the pixel values [33]. This estimates the threshold iteratively and returns two optimal thresholds. The iteration continues until the errors become small or the thresholds no longer change. This iterative multiple threshold process changed RGB regions into binary images which area then processed using morphological closing and opening operations. The unwanted insect pests around the regions, which are smaller than the user-specific threshold, were removed using a size filtering method.

**Fig 4:**
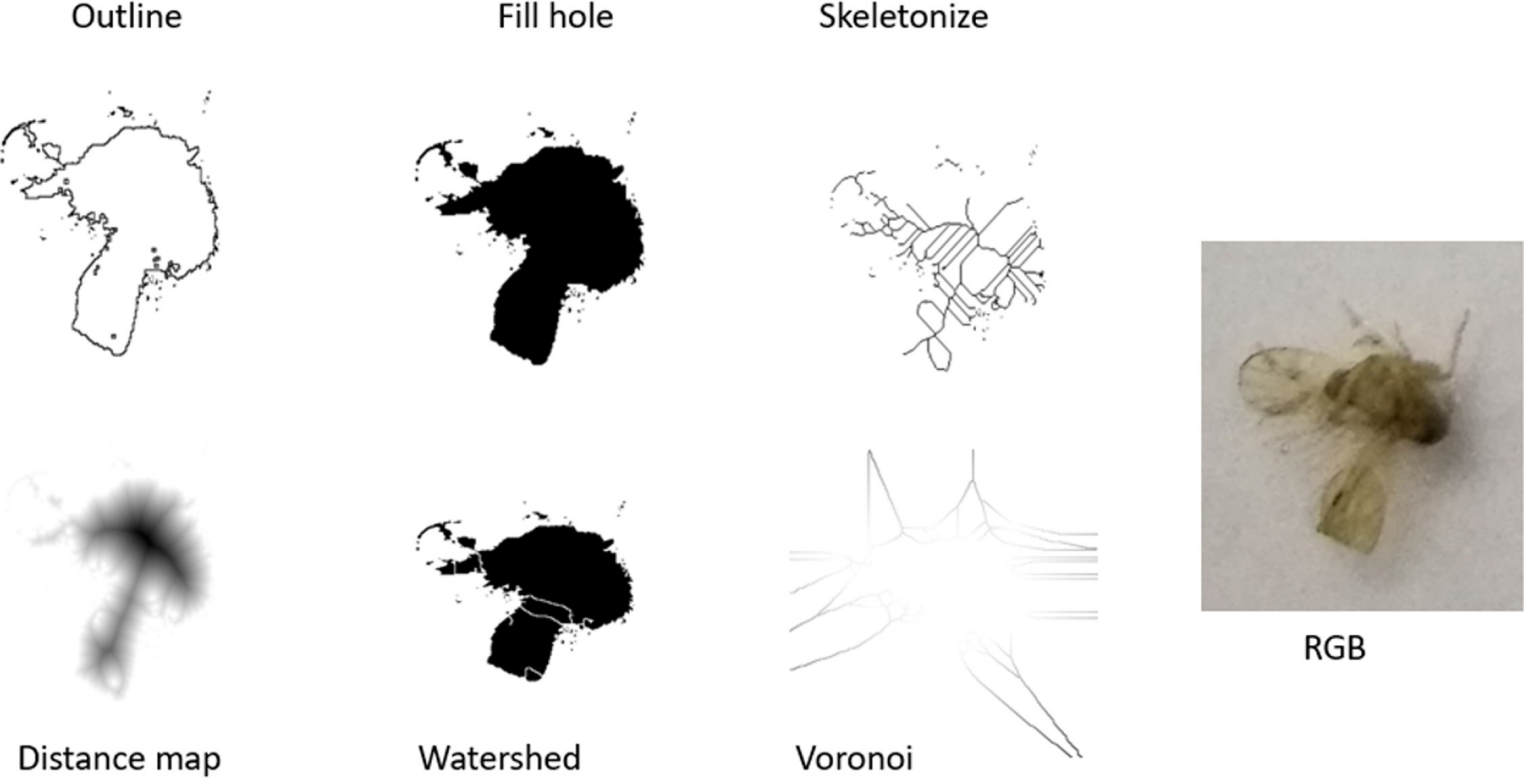
Different types of filtering are applied on captured BP images. These include outline, fill hole, skeletonize, distance map, watershed and Voronoi.

Next, the binary images were transformed by one of the six selected binary operations as shown in Fig 4: outline, fill hole, Skeletonize, Distance Map, Watershed and Voronoi. These filtered images were then input into convolutional neural network (CNN) VGG16 to extract features from the filtered images. The filtered images were then classified as: BPH and benign.

Table 2 presents several proposed pre-processing and CNN architecture pipelines for PENYEK. The selection of colour and filter affects the feature produced by CNN VGG16 architecture. For example, image model A uses grayscale images and these images are filtered by Outline operation. The outline grayscale image is shown in Fig 4. Model B, C, D, E and F use Fill holes, Skeletonize, Watershed, Voronoi and Euclidean distance map, respectively, as shown in Fig 4. An intact RGB image is used without any filter is operated by model G.

**Table 2:** Details of a combination of system components.

Image model	Colour	Filter
*A*	*Grayscale*	*Outline*
*B*	*Grayscale*	*Fill holes*
*C*	*Grayscale*	*Skeletonize*
*D*	*Grayscale*	*Watershed*
*E*	*Grayscale*	*Voronoi*
*F*	*Grayscale*	*Euclidean Distance Map*
*G*	*RGB*	*-*

### CNN architecture

The CNN architecture typically consists of three different layers: convolutional layer, pooling layer and a fully connecter layer.

#### Convolutional layers

This layer consists of kernels (filters) which slide across the insect image. A kernel is the matrix to be convolved with the input image and stride length controls how much the filter convolves across the input image. This layer performs the convolution on the input image with the kernel using Eq (1).

$$y_{k}=\sum_{n=0}^{N-1}x_{n}h_{k-n}$$ (1)

where *x* is pixel, *h* is filter, and *N* is the number of elements in *x*. The output vector is *y*. The subscripts denote the nth element of the vector and *k* is the current kernel.

#### Pooling layers

This down-sampling layer reduces the dimension of output neurons from the convolutional layer to lessen the computational intensity and prevent the overfitting. The max-pooling operation is used in this study. Max-pooling operation selects only the maximum value in each feature map and consequently reducing the number of output neurons.

#### Fully connected layers

This layer has full connection to all the activations in the previous layer.

Table 3 is the summary that details 7 different CNN structures in the observation of the behaviour of variation in model characteristics by training them from scratch (i.e. randomly initializing the layers) on representative filter operation subsets of the whole insect pest image data for BPH classification. This empirical analysis acts as a pilot study, as it allows to establish the feasibility of deep learning methods for the described image analysis problems. Considering a high visual complexity of imperfect BPH images. For example, CNN model 1 has 6 number of layers, 2 number of convolutional layers, (3,3) kernel sizes, (16,16) numbers of feature maps, (3,3) kernel sizes for pooling layer and (128,3) number of fully connected layer outputs. This step is to investigate which filter (Fig 4) performs better in BPH classification to be included in PENYEK pipeline.

**Table 3:** Details of CNN architectures for BN classification on sticky pad datasets.

*CNN structure number*	*Total number of layers*	*Number of convolutional layers*	*Kernel sizes (convolutional layers)*	*Number of feature maps*	*Kernel sizes (pooling layers)*	*Number of fully connected layer outputs*
*1*	*6*	*2*	*3*,*3*	*16*,*16*	*3*,*3*	*128*,*3*
*2*	*6*	*2*	*7*,*3*	*16*,*16*	*2*,*2*	*156*,*4*
*3*	*7*	*2*	*9*,*9*	*16*,*16*	*3*,*3*	*128*,*128*,*3*
*4*	*7*	*2*	*7*,*5*	*16*,*16*	*2*,*2*	*256*,*128*,*3*
*5*	*9*	*3*	*7*,*5*,*3*	*24*,*16*,*16*	*2*,*2*,*2*	*256*,*128*,*3*
*6*	*10*	*4*	*9*,*7*,*5*,*3*	*32*,*128*,*128*,*128*	*3*,*3*,*3*	*2048*,*2048*,*3*
*7*	*11*	*5*	*11*,*5*,*3*,*3*,*3*	*96*,*256*,*384*,*384*,*256*	*3*,*3*,*3*	*4096*,*4096*,*3*

The chosen binary operator is combined with CNN VGG16 architecture to form a complete PENYEK pipeline. In this last step, the binary operator + CNN VGG16 trained on a large volume of annotated data is used. In other words, this study uses VGG16 architecture instead of training a custom CNN from scratch for the task of BPH classification. VGG16 architecture is one of the transfer learning approaches in deep learning that has several advantages such as the architecture’s weights and biases have been trained over millions of images to classify thousands of object and classes. This makes the weights and biases connecting its neurons have been optimally calculated. As the used dataset is small in nature, training VGG16 on a small dataset greatly affects the VGG16’s ability to generalize, often result in overfitting.

To overcome aforementioned problems in training CNN, this research uses fine-tuning technique using the following strategies:

- Use a smaller learning rate,
- Truncate the last layer from 1000 classes to 2 classes, and
- Freeze the weights of the first few layers.

**Fig 5:**
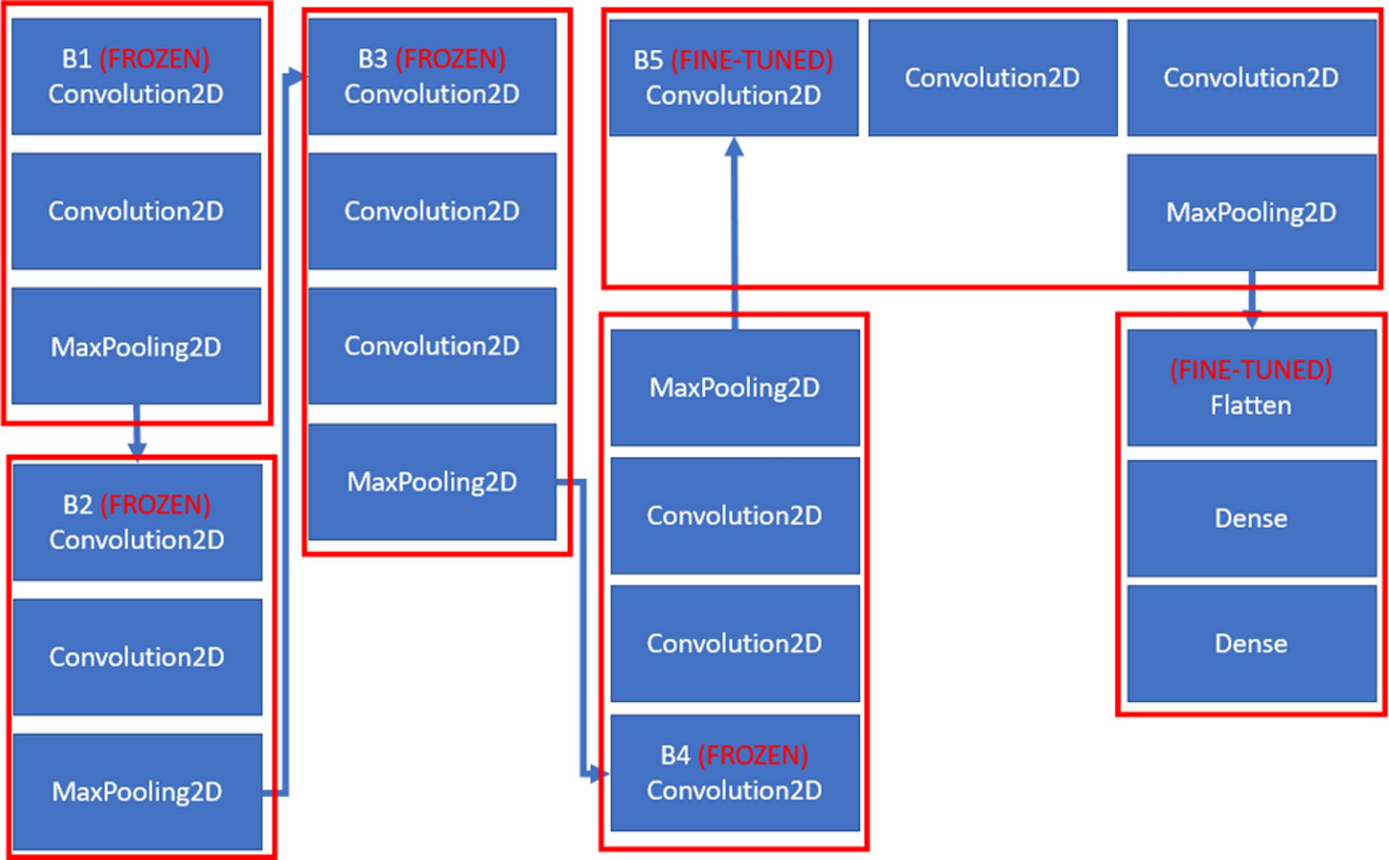
VGG architecture.

The VGG16 architecture that performs these strategies is shown in Fig 5. In general, VGG16 architecture is a 16-layer network. A new feature with the main feature of this architecture was the increased depth of the network. The input images have the size of 224x224 are passed through 5 blocks of convolutional layers where each block is composed of increasing numbers of 3x3 kernels. The stride is fixed to 1 while the convolutional layer inputs are padded such that the spatial resolution is preserved after convolution. The blocks are separated by max-pooling layers. Max-pooling is performed over 22 windows with stride 2. The 5 blocks of convolutional layers are followed by three fully-connected layers. The final layer is a soft-max layer that outputs class probabilities.

### Experiments

The CNN structures and VGG16 architecture are evaluated based on the intact/imperfect insect pest image patches. These image patches contain 2 distinct categories: positive and negative.

Positive patches are derived from manually labelled bounding circle, where each one represents a BPH. Fig 2A shows the positive patches. Negative patches contain most of the uninteresting “negative” areas and benign insects as shown in Fig 2B. To get the uninteresting “negative” areas, this study applies the Canny edge detector to find patches, those that do not contain any BPH. To make the classifier more discriminative, a bootstrapping approach was performed to find useful training patches.

In machine learning, the larger the dataset, the better the generalization performance. In this study, the amount of training data, which is represented by the number of training patches, is much smaller than standard small-scale image classification datasets frequently used by the deep learning field. Therefore, data augmentation was performed to increase the number of images for training, and incorporated invariance to basic geometric transformations into the classifier.

## Results and discussion

This section quantitively describes the results of the PENYEK performance and then discusses qualitative visual results.

### Quantitative

The first experiment is a pilot study to evaluate binary filtering operations: Outline, Fill holes, Skeletonize, Watershed, Voronoi and Euclidean distance map. Seven different CNN structures were devised to create a novel pipeline for identification and classification of heterogenous BPH to test the filters. Table 4 shows the empirical results of the pilot study. On observing the classification performance using the test accuracies, the red highlighted CNN architecture with EDM achieves favourable results on the small representative dataset. Based on empirical analysis and insight to accurately model the characteristics of BPH images, EDM is selected for further evaluation with CNN VGG16 for BPH classification.

**Table 4:** Details of empirically evaluated CNN structures for BPH classification on sticky pad images. 5 cross-validation (CV).

Image Model and CNN structures	Validation average accuracy (BPH classification)	Test average accuracy (BPH classification)
A (1,2,3,4,5,6,7)	0.4235	0.4333
B (1,2,3,4,5,6,7)	0.6208	0.6140
C (1,2,3,4,5,6,7)	0.7025	0.6417
D (1,2,3,4,5,6,7)	0.7	0.6432
E (1,2,3,4,5,5,6,7)	0.6951	0.5376
F (1,2,3,4,5,6,7)	0.7056	0.7571
G (1,2,3,4,5,6,7)	0.4948	0.4948

PENYEK pipeline is shown in Fig 3 in which the pipeline combines EDM and VGG16. VGG16 architecture accepts 224x224 RGB images as input, but in this study, the grayscale image with the same size is fed to the architecture. Table 5 shows the performance of PENYEK by varying the parameters. PENYEK shows promising performance with 95% accuracy, 94% sensitivity, 92% specificity and 93% AUC. This is true when VGG15 is fine-tuned and accepts 224x224 image size as input. However, the performance of VGG16 deteriorates when the architecture is learnt from scratch with 90% accuracy, 90% sensitivity, 83% specificity and 88% AUC. Reducing the size of images further decreases the performance of VGG16 as shown in Table 5. Overall, PENYEK achieves better performance than random performance.

**Table 5:** Performance of VGG16. 5 CV.

Method	Training	Accuracy (%)	Sensitivity (%)	Specificity (%)	AUC (%)
224x224 + EDM + VGG16	Transfer learning	95	94	92	93
224x224 + EDM + VGG16	From scratch	90	90	83	88
21X21 + EDM + VGG16	Transfer learning	78	73	81	82
49x49 + EDM+ VGG16	Transfer learning	79	75	80	85

Table 6 shows the effect of data augmentation on the proposed pipeline performance, this study performs experiments on the VGG16 with input size 224x224 by either using (1) both rotational and translational augmentation; (2) only rotational augmentation; (3) only translational augmentation; and (4) no augmentation. The empirical results show that both translational and rotational augmentations improved the performance compared to no augmentation at all. The accuracy of the data augmentation is 95% while the AUC is 93% and the accuracy of 71% and AUC of 78% for no augmentation.

**Table 6:** Effectiveness of data augmentation. 5 CV.

Method	Accuracy (%)	AUC (%)
VGG16 + Trans & Rot Aug	95	93
VGG16 + Rot Aug	92.6	91
VGG16 + Trans Aug	88.9	91
VGG16 + No Aug	71.3	78.2

Eventually, the performance of PENYEK pipeline is compared with state-of-the-art approaches for image analysis in digital pest identification and classification. These include handcrafted texture and colour descriptors such as the GLCM features, Gabor filter-bank features, LBP histograms, gray histograms, HSV histograms and RGB histograms followed by random forest machine learning. The average classification accuracy and AUC using the state-of-the-art methods is shown in Table 7 for BPH classification. Overall, the accuracy and AUC of PENYEK outperform the state-of-the-art methods.

**Table 7:** Average classification accuracy and AUC of applied methods for BPH classification.

Method	Training	Accuracy (%)	AUC (%)
GLCM + random forest	From scratch	69.93	69.42
Gabor filter + random forest	From scratch	66.27	68.56
LBP histograms + random forest	From scratch	65.79	63.48
Gray histograms + random forest	From scratch	71.55	75.04
HSV histograms + random forest	From scratch	71.62	75.46
RGB histograms + random forest	From scratch	75.67	77.41

### Qualitative

**Fig 6:**
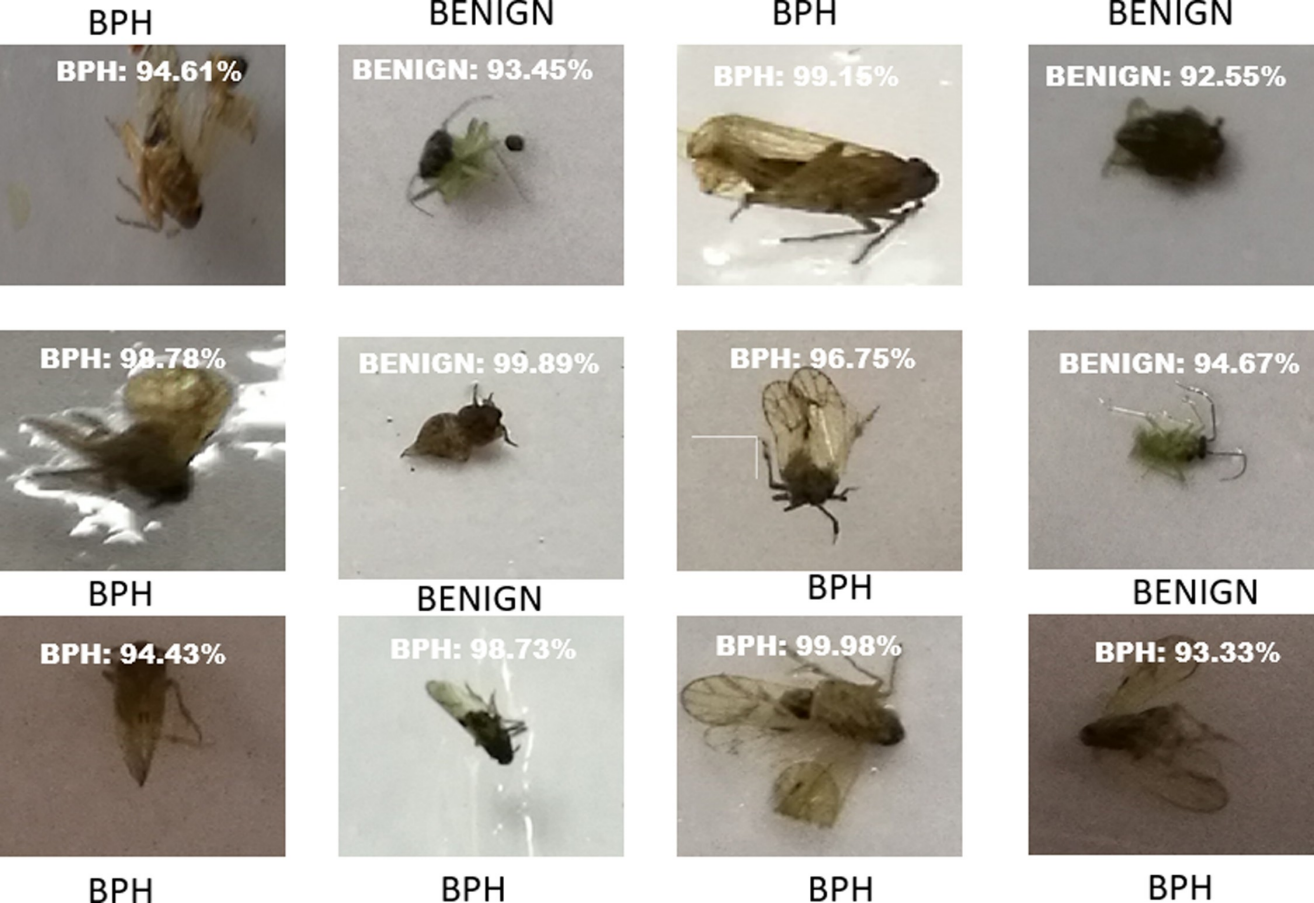
Classification results of 12 test images fed into VGG16. The image size is 244x244 in RGB image. BPH and benign insect pests are classified using the PENYEK pipeline at the specified confidence percentage.

Fig 6 shows an example of the proposed pipeline in operation. In Fig 6, all images in the figure are correctly classified.

## Conclusion

Accurate insect pest detection is very important in agriculture for the estimation of pest population density and dynamics in fields which allows for precision pesticide application. Due to the complex environment background of living pests, it is a big challenge to automatically identify them by image processing. The major challenge in the state-of-the-art automated system is to identify imperfect images. To replace human expertise and to overcome the aforementioned major challenges in the automated system, this study proposed an automated detection pipeline for Brown Planthopper in paddy fields called PENYEK. The PENYEK pipeline leveraged the architecture of VGG16 and Euclidean Distance Map (EDM) by applying the pre-trained weights and biases for classifying imperfect images. VGG16 network pre-trained on the large ImageNet dataset is fine-tuned to learn features of the BPH image dataset. The VGG16 architecture learned to identify BPH based only on positive and negative training samples. The insect pest images are in grayscale and achieves lower accuracy in RGB.

The first component of the proposed pipeline is image processing by applying binary filtering operations and other pre-processing techniques on image patches. From the performance of several CNN structures, EDM shows the best performance of all in term of accuracy. The second component is the VGG16 pre-trained architecture that has been fine-tuned to be trained in a small dataset. The performance of VGG16 architecture increases when fed with EDM images in term of accuracy, sensitivity, specificity and AUC. Moreover, the VGG16 architecture outperforms the state-of-the-art methods in image analysis in term of accuracy and AUC. All in all, qualitative and quantitative empirical results demonstrate the effectiveness of PENYEK pipeline on an insect pest image dataset.
